# Treating traumatic brain injury at sea: how to improve the skills and capabilities of the naval medical personnel

**DOI:** 10.1186/s40779-022-00428-6

**Published:** 2022-12-05

**Authors:** Xin-Jie Hong, Kai-Wei Han, Rong-Bin Chen, Liang Zhao, Dan-Feng Zhang, Lei Jiang, Yi-Ming Li, Li-Quan Lv, Li-Jun Hou, Tao Xu

**Affiliations:** Department of Neurosurgery, Changzheng Hospital, Naval Medical University, Shanghai, 200003 China

**Keywords:** Skill set, Competencies, Traumatic brain injury, Navy

Dear Editor,

Recent advances in military technology have led to the development of sophisticated and intelligent weapons, which increase mortality and morbidity. Since the advent of high-explosion weapons, “shock wave” has surpassed “shrapnel” and become the most important injurious component in conventional combat. Traumatic brain injury (TBI) is a leading cause of disability and death among military personnel and civilians during wartime [1]. Clinically, its symptoms and indications vary depending on the location and severity of the injury. The most frequently observed symptoms included altered consciousness and local dysfunction. To avoid additional injuries and associated mental stress, early diagnosis and treatment are necessary. Moderate and severe TBI worsens if appropriate treatment is not taken promptly. It has earlier been reported that debridement decompression performed within 5.33 h of injury greatly reduces postoperative mortality of patients [1]. Thus, appropriate TBI treatment should be implemented promptly to save the patients’ lives.

In recent decades, the occurrence of TBI in modern maritime combat has been on the rise [2]. Unlike land, it is difficult to provide timely and adequate expert assessment and treatment to TBI casualties in maritime environments and extreme war conditions [3]. In addition, interventions such as neurosurgical treatment are difficult to implement promptly and accurately in frontier war zones. Moreover, long-term deployment to distant maritime operations, insufficient professional medical equipment, and limited experience of front-line medical personnel increase the difficulty of treating naval war wounded. Therefore, researchers from different countries try to find effective strategies to reduce the morbidity and mortality associated with TBI at sea. Of particular concern is the development of modular neurosurgical treatments that can be initiated immediately after injury [4]. Adequate and timely neurological examination, treatment, and monitoring should be carried out throughout the evacuation period. To achieve this aim, naval surgeons and other medical personnel should have appropriate neurosurgical skills before deployment.

The “core of front-line TBI treatment” should be the early detection and management of intracranial pressure (ICP). Uncontrolled ICP progressively causes neurological dysfunction, herniation, and increases the risk of intraoperative mortality. Patients with intracranial hypertension are often treated conservatively with mannitol. Head elevation and hyperventilation may also be useful in such patients. If ICP continues to rise, surgical decompression should be performed. In addition, injured patients should promptly adopt the latest neurosurgical techniques, including external ventricular drainage and decompressive craniectomy.

More importantly, providing immediate care to TBI casualties is essential to reduce secondary brain injury and improve prognosis. In this study, we established a modular procedure for ongoing neurosurgical care for TBI (Fig. 1). The members of the medical service team formed the neurosurgical rescue chain, and each member fulfilled their competent roles and responsibilities. As such, members should be trained during their internships and continuously allowed to attend regular refresher or intensive training programs before deployment. Currently, highly specialized surgical techniques and neurosurgical skills are generally considered to be beyond general surgical education. Therefore, comprehensive training on required skills is advocated, and large hospitals with the necessary facilities, teaching staff, and clinical resources should be involved in training. This approach will yield enough technicians to treat casualties with TBI at sea. Our opinion is that they should aim to gain and continuously improve surgical competence. Regular training sessions using simulation programs can also be used to refresh these skills. These training programs will provide medical personnel with the skills needed to assess neurosurgical emergencies that occur in the ocean.

**Fig. 1:**
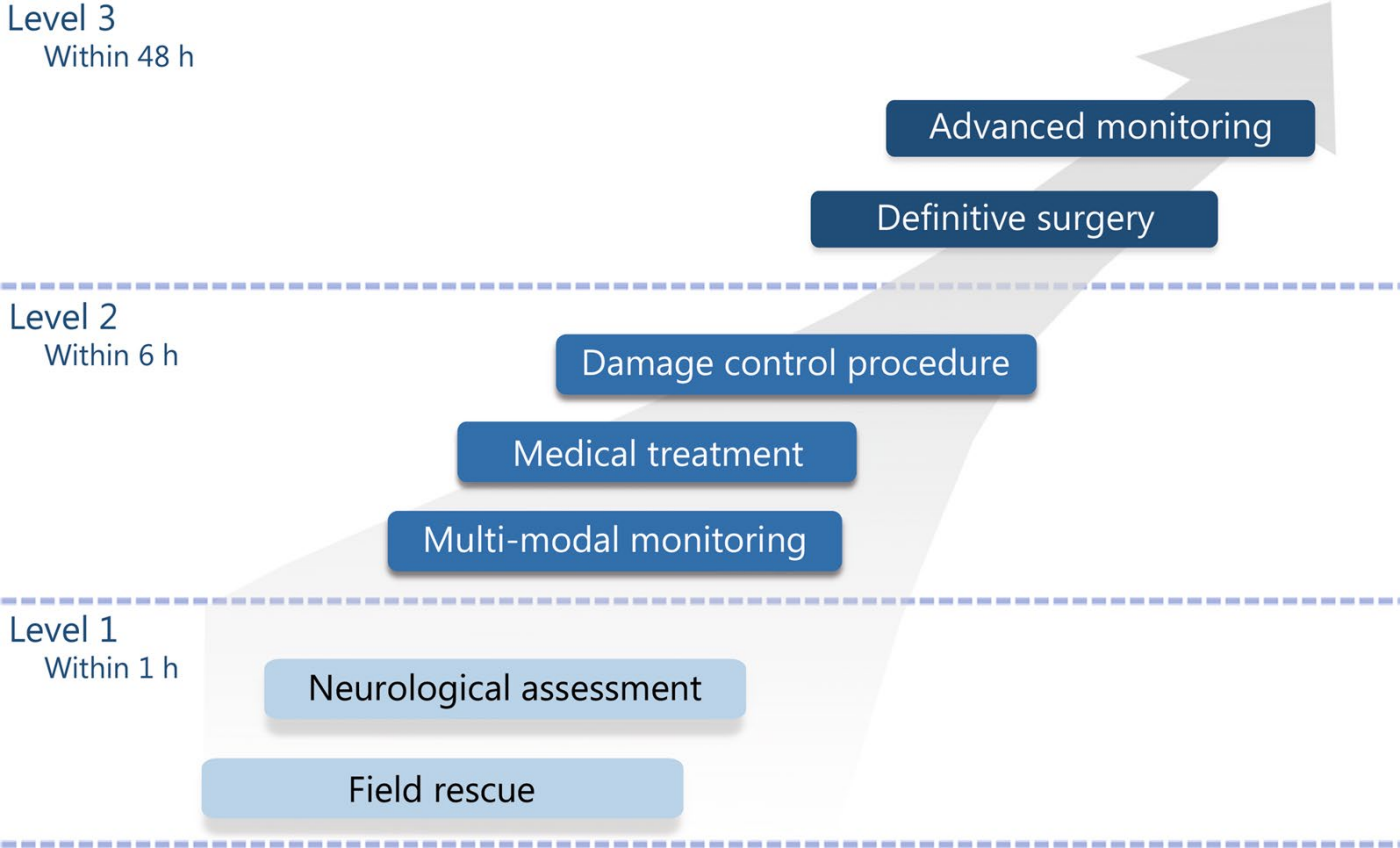
Modular process of continuous neurosurgical treatment for TBI. Level 1: the skills need to be mastered by all naval medical personnel and should be finished within the “golden 1 h” after injury. Level 2: the skills need to be mastered by all naval surgeons (despite their specialty) and better be finished within 6 h after injury. Level 3: the skills need to be mastered by deployed neurosurgeons and should be finished within 48 h after injury. TBI traumatic brain injury

We hope that the introduction of such skills and training methods would help naval medical professionals prepare for and make better treatment decisions for TBI at sea.

## Data Availability

Not applicable.

## Abbreviations

TBI: Traumatic brain injury
ICP: Intracranial pressure
